# A Systematic Review of the Efficacy and Safety Experience Reported for Sorafenib in Advanced Renal Cell Carcinoma (RCC) in the Post-Approval Setting

**DOI:** 10.1371/journal.pone.0120877

**Published:** 2015-04-01

**Authors:** Mayer N. Fishman, Jin Tomshine, William J. Fulp, Pamela K. Foreman

**Affiliations:** 1 H. Lee Moffitt Cancer Center and Research Institute, Tampa, Florida, United States of America; 2 Blue Ocean Pharma LLC, Annandale, New Jersey, United States of America; Seoul National University, KOREA, REPUBLIC OF

## Abstract

**Background:**

Sorafenib was FDA approved in 2005 for treatment of renal cell carcinoma (RCC) based on the results of the pivotal phase 3 clinical trial, TARGET (Treatment Approaches in Renal Cancer Global Evaluation Trial). Since that time, numerous clinical studies have been undertaken that substantially broaden our knowledge of the use of sorafenib for this indication.

**Methods:**

We systematically reviewed PubMed, Web of Science, Embase, Cochrane Library, and www.clinicaltrials.gov for prospective clinical studies using single agent sorafenib in RCC and published since 2005. Primary endpoints of interest were progression-free survival (PFS) and safety. PROSPERO International prospective register of systematic reviews #CRD42014010765.

**Results:**

We identified 30 studies in which 2182 patients were treated with sorafenib, including 1575 patients who participated in randomized controlled phase 3 trials. In these trials, sorafenib was administered as first-, second- or third-line treatment. Heterogeneity among trial designs and reporting of data precluded statistical comparisons among trials or with TARGET. The PFS appeared shorter in second- vs. first-line treatment, consistent with the more advanced tumor status in the second-line setting. In some trials, incidences of grade 3/4 hypertension or hand-foot skin reaction (HFSR) were more than double that seen in TARGET (4% and 6%, respectively). These variances may be attributable to increased recognition of HFSR, or potentially differences in dose adjustments, that could be consequences of increased familiarity with sorafenib usage. Several small studies enrolled exclusively Asian patients. These studies reported notably longer PFS than was observed in TARGET. However, no obvious corresponding differences in disease control rate and overall survival were seen.

**Conclusions:**

Collectively, more recent experiences using sorafenib in RCC are consistent with results reported for TARGET with no marked changes of response endpoints or new safety signals observed.

## Introduction

Sorafenib was approved in 2005 for treatment of renal cell carcinoma (RCC) based on the results of the pivotal phase 3 clinical trial, TARGET (**T**reatment **A**pproaches in **R**enal Cancer **G**lobal **E**valuation **T**rial) [1]. This randomized, double-blind, placebo-controlled, multicenter study examined overall survival (OS), median progression-free survival (PFS), objective response rate (ORR), and safety in 903 patients with histologically confirmed metastatic clear-cell RCC who had had progression after one systemic treatment within the previous 8 months. Patients with brain metastases or prior exposure to vascular endothelial growth factor (VEGF) pathway inhibitors were excluded. In TARGET, sorafenib significantly extended median PFS from 2.8 months in the placebo group to 5.5 months (hazard ratio for disease progression in the sorafenib group, 0.44; 95% confidence interval [CI], 0.35 to 0.55;*P*<0.01), with an acceptable safety profile [1].

While sorafenib was under review by the US Food and Drug Administration (FDA), several expanded access programs were established. In the open-label EU-ARCCS (**EU**ropean **A**dvanced **R**enal **C**ell **C**arcinoma **S**tudy; N = 1150), NA-ARCCS (**N**orth **A**merica-**A**dvanced **R**enal **C**ell **C**arcinoma **S**tudy; N = 2504), and AUS1 (N = 47) studies, median PFS (95% CI) was 6.6 (6.1–7.4) [2], 5.5 (5.1–5.8) [3], and 6.5 (2.61–10.41) [4] months, respectively (**Table 1**). Notably, in these trials, sorafenib was used as a first-line systemic agent in 33%-50% of patients, and patients were not required to have clear cell histology.

**Table 1:** TARGET and expanded access trials.

	NCT00073307TARGET [1]	NCT00492986EU-ARCCS [2]	NCT00111020NA-ARCCS [3]	AUS1 [4]
Trial Design	Randomized Double-blind	Expanded Access	Expanded Access	Expanded Access
N	903	1159	2515	47
Sorafenib arm, n efficacy	451	1150	2504	47
Sorafenib arm, n safety	451	1145	2504	47
**Patient baseline characteristics** ^a^				
Age, median (range),years	58 (19–86)	62 (18–84)	63 (13–93)	60-(34–83)
Male, n (%)	315 (70)	858 (75)	1734 (69)	35 (75)
Race, n (%)				
White	NR	NR	2231 (89)^b^	NR
Black	NR	NR	102 (4)	NR
Hispanic	NR	NR	85 (3)	NR
Asian	NR	NR	38 (2)	NR
Other	NR	NR	96 (4)	NR
ECOG PS, n (%)				
0	219 (49)^c^	460 (40)^d^	NR^e^	14 (30)
1	223 (49)	516 (45)	NR	24 (51)
2	7 (2)	169 (15)	NR	9 (19)
RCC histology, n (%)				
Clear cell	894 (99)	909 (79)	2302 (92)	33 (70)
MSKCC score, n (%)				
Favorable	233 (52)	NR	NR	5 (11)
Intermediate	218 (48)	NR	NR	28 (60)
Poor		NR	NR	14 (29)
Prior systemic therapy, n (%)	903 (100)	765 (67)	1250 (50)	24 (51)
Prior nephrectomy, n (%)	422 (94)	1020 (89)	2081 (83)	37 (79)
**Sorafenib treatment**				
Median (range) duration of treatment, months	5.3	NR	2.8 (<1–18.7)	NR
**Efficacy**				
OS, months (95% CI)	17.8 (NR)	NR	12 (11.0–12.6) 1st line; 10.6 (9.9–13.8) previously treated	11.9 (4.99–18.88)
HR vs PBO(95% CI)	0.88 (0.74–1.04), P = .146;[0.78 (0.62–0.97), P = .029 crossover patients censored]	NA	NA	NA
PFS, months (95% CI)	5.5 (NR)	6.6 (6.1–7.4)	5.5 (5.1–5.8)	6.5 (2.61–10.41)
Response, n(%)				
CR	1 (< 1)	1 (<1)	1 (<1)^f^	1 (2)
PR	43 (10)	45 (4)	67 (4) ^f^	6 (13)
SD	333 (74)	633 (60)	1511 (80) ^f^	29 (62)
**Safety, n (%)**				
Treatment-related AEs	392 (87)	1072 (94)	NR	NR
Grade 3 or 4	132 (29)	519 (45)	NR	NR
Treatment-emergent AEs	NR	NR	NR	44 (94)
Grade 3 or 4	NR	NR	NR	28 (60)
SAEs	154 (34)	515 (45)	NR	10 (21)^g^
Treatment-emergent AEs (n%)				
Fatigue	165 (37)	388 (34)^h^	NR	20 (43)
Grade 3 or 4	22 (5)	81 (7)	113 (5) ^g^	4 (9)
HFSR	134 (30)	645 (56)		25 (53)
Grade 3	25 (6)	149 (13)	238 (10)	6 (13)
Rash or desquamation	180 (40)	379 (33)	NR	22 (47)
Grade 3 or 4	4 (1)	60 (5)	124 (5)	3 (6)
Alopecia	122 (27)	375 (33)	NR	10 (21)
Grade 3 or 4	1 (<1)	0	2 (<1)	0
Nausea	102 (23)	198 (17)	NR	19 (40)
Grade 3 or 4	3 (< 1)	14 (1)	38 (1)	4 (9)
Diarrhea	195 (43)	633 (55)	NR	15 (32)
Grade 3 or 4	11 (2)	84 (7)	58 (2)	0
Hypertension	76 (17)	223 (20)	NR	11 (23)
Grade 3 or 4	16 (4)	70 (6)	114 (5)	4 (9)
Weight loss	46 (10)	128 (11)	NR	NR
Grade 3 or 4	3 (< 1)	13 (1)	4 (<1)	NR
Reduced appetite/anorexia	NR	249 (22)	NR	16 (34)
Grade 3 or 4	NR	33 (3)	26 (1)	1 (2)
Dyspnea	65 (14)	NR	NR	NR
Grade 3 or 4	16 (4)	NR	NR	NR
Constipation	68 (15)	81 (7)	NR	NR
Grade 3 or 4	3 (1)	3 (<1)	4 (<1)	NR
**Additional References**		[5–15]	[16–21]	[22–24]
**Funding**	Bayer HealthCare and Onyx Pharmaceuticals	Bayer HealthCare and Onyx Pharmaceuticals	Bayer HealthCare and Onyx Pharmaceuticals	Bayer Australia

Abbreviations: AE = adverse event; CI = confidence interval; CR = complete response; ECOG PS = Eastern Cooperative Oncology Group performance status; HFSR = hand-foot skin reaction; HR = hazard ratio; MSKCC = Memorial Sloan Kettering Cancer Center; NE = estimable; NR = not reported; OS = overall survival; PBO = placebo; PFS = progression-free survival; PR = partial response; SAE = serious adverse event; SD = stable disease.

^a^ Unless otherwise specified, refers to entire study population.

^b^ Data missing for 36 pts.

^c^ Data missing for 2 pts.

^d^ Data missing for 5 pts.

^e^ Eligibility criteria included ECOG PS 0–2 with waivers granted to selected pts with PS 3 or 4.

^f^ n = 1891.

^g^ Considered treatment related.

^h^ All reported AEs were considered treatment related.

The TARGET trial, which formed the basis for sorafenib approval, and these expanded access studies, represented the collective experience with sorafenib in RCC at the time of its approval. Since then, the treatment landscape for RCC has changed considerably. Clinicians now have nearly 10 years of additional experience in the use of sorafenib, managing its side effects, and evaluating response to angiogenesis inhibitors in larger and more diverse patient populations. Moreover, additional targeted systemic therapies, such as sunitinib, axitinib, dovitinib, bevacizumab, trebananib, and temsirolimus, pazopanib, and everolimus have been, and continue to be, investigated in RCC. An increasing emphasis is being placed on the use of these agents in the first-line setting, and several clinical trials have focused on head-to-head comparisons with sorafenib. Since complete objective responses are rare, studies are also investigating the use of sorafenib sequentially (either prior to, or following) or in combination with other targeted agents, as an open-ended management of metastatic kidney cancer.

The abundance of data from published clinical studies of sorafenib in RCC substantially broadens our knowledge base. To date, the collective experience has not been comprehensively reviewed and aggregated. The objective of this study is to understand the body of evidence defining a more contemporary perspective on efficacy and safety of sorafenib in patients with RCC treated since 2005. The issue of relative efficacy versus comparator drugs is not addressed in this review.

## Methods

### Protocol registration

The protocol for this study is registered at the International Prospective Register of Systematic Reviews (PROSPERO) and may be accessed at http://www.crd.york.ac.uk/PROSPERO/display_record.asp?ID=CRD42014010765#.U9gsn_ldW3A.

### Databases, search methodology, and eligibility criteria

The search terms (“sorafenib” or “Nexavar” or “BAY 43–9006”) AND (“RCC” or “renal cancer” or “kidney cancer”) were used to identify relevant publications, including meeting abstracts, in 4 electronic databases: PubMed (1/1/2005–3/3/2014), ISI Web of Science (1/1/2005–2/28 2014), Embase (1/1/2005–3/10/2014), and Cochrane Library (1/1/2005–2/28/2014). Bibliographies from pertinent review articles were hand-searched for additional relevant citations. Two independent reviewers examined titles and abstracts to determine eligibility for all identified records. When eligibility could not be determined from the abstract, the full publication was used. Disagreements were resolved by discussion between the two reviewers. Records were excluded for the following reasons in this sequence: 1) review articles, meeting reports, editorials, or guidelines; 2) not written in English; 3) reported only preclinical data, phase 1 trial data, data from a pilot or exploratory study, or data from a study with <20 patients receiving sorafenib; 4) reported data for patients included in the TARGET clinical trial; 5) reported data from a trial in which there was no discrete sorafenib arm or sorafenib was used only in combination with another systemic anticancer agent; 6) results from the reported study did not include patients with RCC, or the results in patients with RCC were only presented pooled with other tumor types; 7) presented results from a retrospective or observational study. Following identification of eligible studies, Internet searches (using Google) were performed to identify additional publications. Search terms consisted of the last names of the first and last authors for each of the already identified publications.

In addition, www.clinicaltrials.gov was searched, using the same search terms, to identify clinical trials registered between 1/1/2005 and 5/12/2014. Trials were excluded if they 1) were phase 1 or included fewer than 20 patients receiving sorafenib; 2) had no discrete sorafenib arm, or sorafenib was used only in combination with another systemic anticancer agent; or 3) were observational.

### Data collection

Data were extracted to spreadsheets that had been pilot-tested to ensure that the included fields encompassed all desired data. When interim and mature data were both identified, the most recent data were used. Data were extracted exclusively for single-agent sorafenib arms. The primary endpoints of interest in this study were PFS and adverse events (AEs), especially hypertension, diarrhea, hand-foot skin reaction, and fatigue. Secondary outcomes of interest were OS and response rate (RR).

One reviewer extracted data and a second reviewed each field for accuracy. In some instances, where data are reported as n (%), only the number of patients or the percentage was published. For consistency of reporting, the corresponding values were calculated based on the total number of patients in the relevant study population. Similarly, when units for duration of treatment, PFS, or OS values were reported in days or weeks, values were converted to months as follows. Days: 12 x (reported value/365); Weeks: 12 x (reported value/52).

The following variables were extracted for each phase 3 and expanded access study (**Tables 1** and **2**): line of therapy; total number of patients; number of patients in the sorafenib arm; brief description of trial design; patient age, gender, race, Eastern Cooperative Oncology Group performance status (ECOG PS), RCC histology, Memorial Sloan Kettering Cancer Center (MSKCC) status; number of prior systemic therapies; prior nephrectomy; duration of sorafenib treatment; OS and hazard ratio, median PFS and hazard ratio, clinical benefit rate (complete response [CR], partial response [PR], stable disease [SD]), treatment-related and treatment-emergent AEs (overall and grade 3/4), and selected AEs that were observed in >20% of patients in any phase 3 study (overall and grade 3/4). For phase 2 and smaller studies, a subset of these variables was extracted (**Table 3** [25–56] and **Table 4** [57–67]).

**Table 2:** Randomized, open-label, phase 3 trials of sorafenib in RCC published following the TARGET trial.

	NCT00920816 AGILE 1051 [68,69]	NCT01481870CROSS-J-RCC^a^ [70]	NCT00732914SWITCH [71]		NCT00678392AXIS [72–75]	NCT00474786INTORSECT [76]	NCT01223027GOLD-RCC [77]	NCT01030783TIVO-1 [78–83]
Line of therapy	1st	1st	1st	2nd^b^	2nd	2nd	3rd	1st or 2nd
Trial design	sorafenib vs axitinib	sorafenib followed by sunitinib at progression and vice versa	sorafenib followed by sunitinib at progression vice versa		sorafenib vs axitinib	sorafenib vs temsirolimus	sorafenib vs dovitinib	sorafenib vs tivozanib
N	288	124	365		723	512	570	517
Sorafenib arm, n efficacy	96	63	182	76	362	253	286	257
Sorafenib arm, n safety	96	63	177	76	355	252	284	257
**Patient baseline characteristics**								
Age, median (range)	58 (20–77)	66 (44–79)	64 (39–84)	65 (40–83)	61 (22–80)	61 (21–80)	62 (18–81)	59 (23–85)
Gender								
Male, n (%)	74 (77)	53 (84)	138 (76)	135(74)	258 (71)	192 (76)	219 (77)	189 (74)
Race								
White	66 (69)	NR	NR	NR	269 (74)	163 (64)	232 (81)	249 (97)
Black	0	NR	NR	NR	4 (1)	NR	5 (2)	0
Hispanic	NR	NR	NR	NR	0	NR	0	0
Asian	24 (25)	NR	NR	NR	81 (22)	50 (20)	40 (14)	8 (3)
Other	6 (6)	NR	NR	NR	8 (2)	40 (16)	9 (3)	0
ECOG PS, n (%)								
0	55 (57)	NR	124 (68)	112 (61)	200 (55)	113 (45)	NR	139 (54)
1	41 (43)	NR	58 (32)	70 (38)	160 (44)	139 (55)	NR	118 (46)
2	0	NR	0	0	0	0	NR	NR
Data missing	0	NR	0	0	NR	1 (<1)	NR	NR
RCC histology, n (%)								
Clear cell	NR (100)	NR	164 (90)	154 (84)	NR (100)	208 (82)	NR (100)	NR (100)
MSKCC score, n (%)								
Favorable	53 (55)	14 (22)	71 (39)	90 (45)	101 (28)	44 (17)	59 (21)	87 (34)
Intermediate	40 (42)	49 (78)	108 (59)	94 (51)	130 (36)	177 (70)	162 (57)	160 (62)
Low/poor	2 (2)	0	1 (<1)	1 (<1)	120 (33)	32 (13)	65 (23)	10 (4)
Missing data	1 (1)	0	NR	NR	11 (3)	0	0	0
Prior systemic therapy								
0	(96) 100^a^	63 (100)^c^	182 (100)	0	0	0	0	181 (70)
1	0	0	0	76 (42)	NR (100)	512 (100)	0	76 (30)
>1	0	0	0	0	0	0	NR (100)	0
Prior nephrectomy	86 (90)	56 (89)	167 (92)	168 (92)	NR	219 (87)	260 (91)	(100)
**Sorafenib treatment**								
Median (range) duration, months	10.0 (0.2–21.2)	NR	Mean (SD): 8.7 (8.7)	Mean (SD): 3.7 (3.5)	5.0 (0.03–20)	3.6 (0.2–24.2)	3.7 (< 1.0–16.9)	9.5 (NR)
**Efficacy**								
OS, months (95% CI)	NR	NR	NR		19.2(17.5–22.3)	16.6(13.6–18.7)	11.0(8.6–13.5)	29.3 (NR)
HR vs comparator (95% CI)	NR	NR	NR		0.969 AX vs SOR (0.800–1.174) *P* = 0.3744	1.31 TEM vs SOR (1.05–1.63) *P* = 0.01	0.96 DOV vs SOR (0.75–1.22)	1.245 TIV vs SOR (0.954–1.624) *P* = 0.105
PFS, months (95% CI)	6.5 (4.7–8.3)	7.0 (NR)	5.9 (NR)	2.8 (NR)	4.7 (4.6–5.6)	3.9 (2.8–4.2)	3.6 (3.5–3.7)	9.1 (7.3–9.5)
HR vs comparator (95% CI)	0.77 AX vs SOR (0.56–1.05);*P* = 0.038	0.67 SU vs SOR (0.42–1.08)	1.19 SO vs SU (<1.47^d^); *P* = 0.92	0.55 SU vs SO (<0.74^d^); *P* = 0.0001	0.665 AX vs SOR(0.544–0.812)	0.87 TEM vs SOR (0.71–1.07) *P* = 0.19	0.86 DOV vs SOR (9.72–1.04); *P* = 0.063	0.797^e^ TIV vs SOR (0.639–0.993) *P* = 0.042
Clinical benefit, n (%)								
CR	0	NR	5 (3) ^i^	1 (1)	0	1 (<1)	0	2 (<1)
PR	14 (15)	NR	50 (28) ^i^	4 (5)	43 (9)	19 (8)	11 (4)	56 (23)
SD	51 (53)	NR	68 (38) ^i^	19 (25)	197 (54)	153 (60)	149 (52)	68 (65)
**Safety, n (%)**								
Treatment-related AEs	NR	NR	NR	NR	NR	NR	NR	214 (83)
Grade 3 or 4	NR	NR	NR	NR	NR	NR	NR	131 (51)
Treatment-emergent AEs^f^	90 (94)	NR	172 (97)	64 (84)	346 (97)	252 (100)	NR	249 (97)
Grade 3 or 4	NR	NR	117 (66)	27 (36)	NR	174 (69)	NR	179 (70)^h^
SAEs	24 (25)	NR	NR	NR	110 (31)	85 (34)	NR	NR
Fatigue	25 (26)	26 (42)^g^	57 (32)	9 (12)	112 (32)	85 (34)	97 (34)	41 (16)
Grade 3 or 4	1 (1)	1 (2)^g^	8 (4.5)	0	18 (5)	18 (7)^h^	24 (8)	9 (4)
HFSR	37 (39)	54 (86)	69 (39)	16 (21)	181 (51)	131 (52)	115 (40)	139 (54)
Grade 3	15 (16)	16 (25)	21 (12)	5 (7)	57 (16)	38 (15)^h^	18 (6)	47 (17)
Rash or desquamation	19 (20)	31 (50)^g^	53 (30)	12 (16)	112 (32)	88 (35)	66 (23)	NR
Grade 3 or 4	1 (1)	9 (15)^g^	3 (2)	1 (1)	14 (4)	8 (3)^h^	6 (2)	NR
Alopecia	18 (19)	NR	55 (31)	4 (5)	115 (32)	78 (31)	61 (21)	55 (21)
Grade 3 or 4	NR	NR	0	0	0	0^h^	1 (< 1)	0
Nausea	14 (15)	6 (10)	39 (22)	6 (8)	77 (22)	71 (28)	83 (29)	19 (7)
Grade 3 or 4	1 (1)	0	2 (1)	1 (1)	4 (1)	3 (1)^h^	7 (2)	1 (<1)
Diarrhea	38 (40)	26 (41)	96 (54)	26 (34)	189 (53)	158 (63)	128 (45)	84 (33)
Grade 3 or 4	5 (5)	4 (6)	9 (5)	3 (4)	26 (7)	14 (6)^h^	11 (4)	17 (7)
Hypertension	28 (29)	28 (44)	57 (32)	6 (8)	103 (29)	NR	79 (28)	88 (34)
Grade 3 or 4	1 (1)	11 (17)	16 (9)	2 (3)	39 (11)	NR	47 (17)	45 (18)
Weight loss	23 (24)	NR	NR	NR	74 (21)	51 (20)	87 (31)	53 (21)
Grade 3 or 4	3 (3)	NR	NR	NR	5 (1)	5 (2)^h^	1 (< 1)	9 (4)
Reduced appetite/ anorexia	18 (19)	25 (40)	37 (21)	12 (16)	101 (29)	93 (37)	99 (35)	24 (9)
Grade 3 or 4	0	0	2 (1)	0	13 (4)	8 (3)^h^	14 (5)	2 (1)
Dyspnea	NR	NR	NR	NR	NR	45 (18)	57 (20)	22 (9)
Grade 3 or 4	NR	NR	NR	NR	NR	11 (4)^h^	21 (7)	5 (2)
Constipation	NR	NR	NR	NR	72 (20)	57 (23)	69 (24)	NR
Grade 3 or 4	NR	NR	NR	NR	3 (1)	1 (< 1)^h^	3 (1)	NR
Hypo-thyroidism	7 (7)	19 (33)^i^	NR	NR	29 (8)	NR	8 (3)	NR^j^
Grade 3 or 4	0	1 (2)^i^	NR	NR	0	NR	0	NR
**Additional References**	[84]		[85,86]	[87–96]		[97,98]	[99–101]	[83,102,103]
**Funding**	Pfizer	Yamagata University	Bayer Pharma AG	Pfizer		Pfizer	Novartis	Aveo Oncology and Astellas

Abbreviations: AE = adverse event; AX = axitinib; CI = confidence interval; CR = complete response; ECOG PS = Eastern Cooperative Oncology Group performance status; DOV = dovitinib; HFSR = hand-foot skin reaction; HR = hazard ratio; MSKCC = Memorial Sloan-Kettering Cancer Center; NR = not reported; OS = overall survival; PFS = progression-free survival; PR = partial response; SAE = serious adverse event; SD = stable disease; SOR = sorafenib; SU = sunitinib; TEM = temsirolimus; TIV = tivozanib.

^a^ Data reported only for first-line sorafenib.

^b^ Unless otherwise noted, baseline characteristics refer to the overall SU-SO population at study entry (n = 176); characteristics of patients who crossed over are NR

^c^ Except adjuvant IFNa.

^d^ 1-sided CI.

^e^ HR for progression of death.

^f^ Occurring in >20% of patients in any phase 3 study.

^g^ Data missing for 1 pt.

^h^ Reported as grade ≥3.

^i^ Data missing for 5 pts.

^j^ 18 (7%) had normal thyroid-stimulating hormone levels prior to dosing that increased to >10 IU/mL after treatment; 5 (2%) had low T3 and 2 (1%) had low T4 on or after the date that the increases in thyroid-stimulating hormone were observed.

**Table 3:** Phase 2 trials of sorafenib in RCC published following the TARGET trial.

	NCT00467025 [25]	NCT00126594 [26]	NCT00117637 [27]	NCT00609401ROSORC [28–30]	NCT00618982 [31,32]	NCT00866320 [33]	NCT00079612 [34]	NCT00661375/ NCT00586495 (extension study) [35]	[36]
Line of therapy	1st	1st	1st	1st	1st	≥2nd	≥2nd^a^	≥2nd	≥2nd
Trial design	Randomized double blind: AMG 386 + sorafenib vs sorafenib + placebo	Randomized open label: sorafenib vs sorafenib + low dose interferon-alpha	Randomized open-label: sorafenib vs interferon-alpha	Randomized open label: sorafenib + interleukin-2 vs sorafenib	Single arm: dose escalation	Single arm: sorafenib in sunitinib or bevacizumab refractory patients	Randomized discontinuation: sorafenib vs placebo in patients refractory to approved therapies	Single arm: sorafenib in patients with nephrectomy and failed cytokine therapy	Single-arm: sorafenib after interleukin-2 + interferon-alpha
N	152	80	189	128	83	47	202	131	41
Sorafenib arm, n efficacy	51	40	97	62	67	47	32	129	36^b^
Sorafenib arm, n safety	50	40	97	62	83	47	202	131	38
Previous systemic therapy, n (%)	0	0	0	0	0	47 (100)	29 (91)	129 (100)	38 (100)
1	0	0	0	0	0	47 (100)	NR	46 (36)	0
>1	0	0	0	0	0	23 (49)	NR	83 (64)	0
Nephrectomy, n (%)	NR	40 (100)	95 (98)	46 (74)	NR	44 (94)	29 (91)	129 (100)	35 (85)
**Efficacy**									
OS, months (95% CI)	27.1 (19.7-NE^d^)	NE^e^	NR	33 (16–43)	NR	16.0 (7.6–32.2)	NR	25.3 (19.0–32.0)	16.6 (NE)
PFS, months (95% CI)	9.0 (5.5–10.9)	7.4 (5.5–9.2)	5.7 (5.0–7.4)	6.9 (3.5–15)	7.4 (6.3–9.7)	4.4 (3.6–5.9)	5.5	7.9 (6.4–10.8)	7.4 (6.5–13.1)
Response rate, (%) [95% CI]	(25)[14–40]	(30) [16.6–46.5]	NR	NR	NR	NR	NR	(19) [13–27]	44 [NR]
CR	1(2)	1 (3)	0 (0)	NR	0	0 (0)	NR	0 (0)	3 (8)
PR	12 (24)	11 (28)	5 (5)	9 (15)	12 (18)	1 (2)^f^	NR	25 (19)	13 (36)
SD	30 (59)	17 (43)	72 (74)	27 (60)	46 (69)	20 (43)	NR	87 (67)	18 (50)
**Safety**									
Treatment-emergent AEs, n (%)	50 (100)	NR	92 (95)^g^	NR	80 (96)	NR	202 (100)^c^	127 (97)^g^	NR
Grade 3 or 4	43 (86)^h^ ^,^ ^i^	NR	40 (41)^g^ ^,^ ^h^	NR	NR	NR	133 (65)	90 (69)^g^	NR
Fatigue	11 (22)	NR	42(43)^g^	10 (16)	45 (54)	26 (58)^g^	147 (73)	22 (17)^g^	NR
Grade 3 or 4	0^h^	10 (25)	5 (5)^g^ ^,^ ^h^	1 (2)^h^	NR	8 (18)	13 (7)	3 (2)^g^	NR
HFSR	27 (54)	NR	58 (60)^g^	32 (52)	54 (65)	31 (79)	125 (62)	76 (58)^g^	19 (46)
Grade 3	14 (28)^h^	10 (25)	11 (11)^g^ ^,^ ^h^	6 (10)^h^	NR	14 (31)	27 (13)	12 (9)^g^	0
Rash or desquamation	15 (30)	NR	40(41)^g^	NR	46 (55)	14 (31)	134 (66)	54 (41)^g^	NR
Grade 3 or 4	4 (8)^h^	2 (5)	6 (6)^g^ ^,^ ^h^	NR	NR	3 (7)	5 (2)	5 (4)^g^	NR
Diarrhea	28 (56)	NR	53 (55)^g^	17 (27)	53 (64)	27 (60)	117 (58)	56 (43)^g^	20 (49)
Grade 3 or 4	4 (8)^h^	13 (33)	6 (6)^g^ ^,^ ^h^	0	NR	4 (9)	8 (4)	7 (5)^g^	2 (5)
Hypertension	23 (46)	NR	22 (23)^g^	10 (16)	40 (48)	16 (36)	86 (43)	43 (33)^g^	15 (37)
Grade 3 or 4	7 (14)^h^	2 (5)	2 (2)^g^ ^,^ ^h^	4 (6)^h^	NR	4 (9)	62 (31)	22 (17)^g^	0
**Additional references**	[37]	[38–40]	[41–44]	[45–48]		[49,50]		[49–55]	[56]
**Funding**	Amgen Incorporated	NCI's Cancer Therapy Evaluation Program	Bayer Healthcare Pharma-ceuticals	Funded in part by Bayer HealthCare	Funded in part by Bayer HealthCare	Bayer, Onyx	Bayer, Onyx	Bayer	Bayer Hispania S.L.

Abbreviations: AE = adverse event; CI = confidence interval; CR = complete response; HFSR = hand foot skin reaction; NE = estimable; NR = not reported; OS = overall survival; PFS = progression-free survival; PR = partial response; SD = stable disease.

^a^ NR for 3 patients.

^b^ Tumor assessment was not possible in five patients and they were excluded from the efficacy analysis.

^c^ Treatment emergent AEs were reported for the total 202 patients, which included both patients randomized to the sorafenib and to the placebo arm after a 12 week run-in period of sorafenib treatment.

^d^ Interim analysis.

^e^ Median overall survival was not reached.

^f^ Unconfirmed.

^g^ All reported AEs were considered treatment related.

^h^ Grade ≥3.

^i^ Including 2 grade 5.

**Table 4:** Additional small trials and patient series in RCC published since the TARGET trial.

Reference	Hermann et al 2008 [57]	Zhang et al 2009 [58]	Imarisio et al 2012 [59]	NCT00586105 [60]	Yang et al 2012 [61]	Sun et al 2008 [62]	Kapoor et al 2008 [63]	Battglia et al 2009; Gernone et al 2009 [64,65]
Line of therapy	≥2nd	2nd^a^	NR	NR	1st or ≥2nd	≥2nd	NR	≥2nd
Trial design	Patient series	Patient series^b^	Patient series	Single-arm	Single-arm	Single-arm	Single-arm	Single-arm
N	40	98	80	39	30	62	21	22
Sorafenib arm, n efficacy	20	39^c^	33^d^	39	30	62	21	20
Sorafenib arm, n safety	20	39^b^	33	39	30	62	21	22
**Prior treatments, n (%)**								
Prior systemic therapy	40 (100)	NR^a^	NR	NR	13 (43)	62 (100)	NR	22 (100)
Nephrectomy	19 (95)	91 (93)	NR	NR	27 (90)	43 (85)	11 (52)	21 (96)
**Efficacy**								
OS, months (95% CI)	NR	NR^e^	NR	7.8 (0.9–13.4)	16 (10.2–21.8)	NR^f^	NR	NR
PFS, months (95% CI)	6.4 (NR)	NR^e^	NR	5.5 (4.1–7.8)	14 (0–31.7)	9.5 (NR)	8.4 (1.2–59)^g^	NR
Response rate, n (%)								
CR	0	NR^e^	NR	0	1 (3)	1 (2)	NR	0
PR	2 (10)	NR^e^	NR	5 (13)	4 (13)	11 (18)	NR	13 (59)
SD	14 (60)	NR	NR	27 (69)	19 (63)	36 (53)	NR	7 (31)
**Safety, n(%)**								
Treatment-emergent AEs	NR	NR	NR	39 (100)	NR	NR	NR	NR
Grade 3 or 4	NR	NR	NR	NR	NR	NR	NR	NR
Fatigue	10 (50)	30 (77)^h^	16 (48)	12 (31)	5 (17)	NR	NR	NR
Grade 3 or 4	1 (5)	2 (5)	4 (12)	NR	0	NR	NR	6 (27)
HFSR	8 (40)	21 (54)^h^	10 (30)	25 (64)	18 (60)	NR	NR	NR
Grade 3	3 (15)	5 (13)	2 (6)	NR	8 (27)	10 (16)	NR	5 (22)
Rash or desquamation	8 (40)	11 (28)^h^	4 (12)	9 (23)	9 (30)	NR	NR	NR
Grade 3 or 4	3 (15)	0	1 (3)	NR	1 (3)	NR	NR	4 (18)
Diarrhea	11 (55)	16 (41)^h^	9 (27)	14 (36)	10 (33)	NR	NR	NR
Grade 3 or 4	2 (10)	0	3 (9)	NR	1 (3)	3 (5)	NR	2 (9)
Hypertension	6 (30)	7 (18)^h^	3 (9)	7 (18)	9 (30)	NR	NR	NR
Grade 3 or 4	2 (10)	1 (<1)	3 (9)	NR	1 (3)	2 (3)	NR	3 (13)
**Additional references identified**	[66]						[67]	
**Funding**	NR	NR	NR	Bayer HealthCare	China Charity Federation	NR	NR	NR

Abbreviations: AE = adverse event; CI = confidence interval; CR = complete response; HFSR = hand foot skin reaction; NR = not reported; OS = overall survival; PFS = progression-free survival; PR = partial response; SD = stable disease.

^a^ All 39 pts for which results are posted received 2nd-line sorafenib.

^b^ Patients received either 1st-line sorafenib (n = 43), 1st-line sorafenib + IFN (n = 16), or 2nd-line sorafenib (n = 39).

^c^ Population who received 2nd-line sorafenib.

^d^ Subset of pts with RCC.

^e^ Results not reported individually for pts receiving single-agent sorafenib.

^f^ Not reached after 278 days mean follow-up.

^g^ Median (range).

^h^ Grade 1–2.

## Results

**Fig 1:**
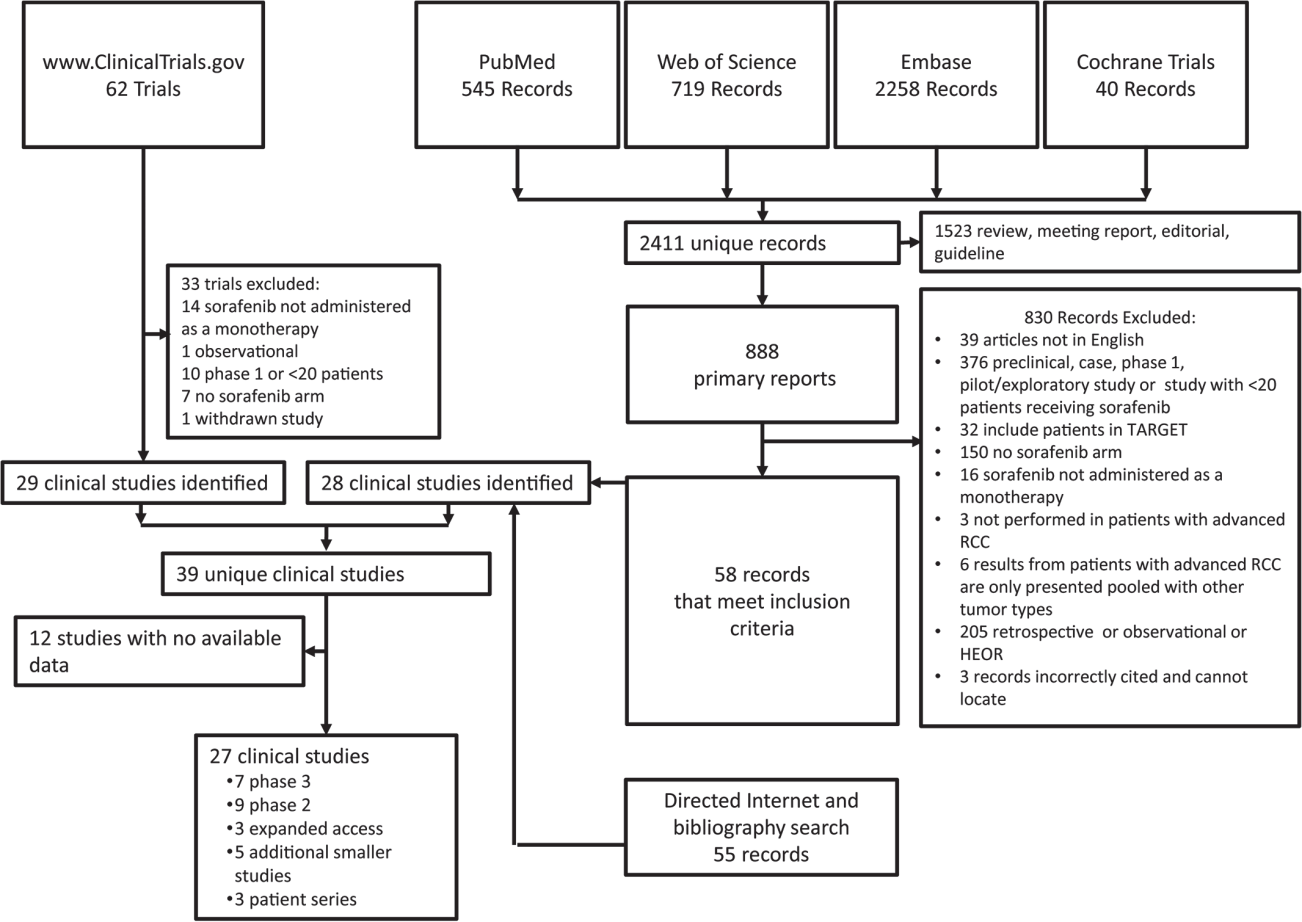
Flow of information. Selection process for included trials.

**Fig. 1** depicts the flow of information informing the selection of clinical studies reviewed. A total of 2411 publications were identified in searches of PubMed, ISI Web of Science, Embase, and Cochrane Library databases. Among these, 888 were primary data reports. Fifty-eight records identified through these databases met inclusion criteria. An additional 55 records meeting inclusion criteria were identified through directed searching of the Internet or bibliographies of review articles. Collectively, these publications identified 28 clinical studies (27 from database searches and 1 from directed searching). An additional 11 clinical studies were identified by searching www.ClinicalTrials.gov. Thirty of the resulting 39 unique identified studies had available results.

### Phase 3 and expanded access trials

Eleven randomized controlled phase 3 trials of sorafenib in RCC have been undertaken since the TARGET trial. Three studies were excluded from this review because data are not yet available (expected in 2016) [104–107]. One additional trial was identified as a phase 3 trial, but it enrolled only 39 patients and was not a controlled trial [60]. Results for this trial are therefore considered below in the context of other small trials and are included in **Table 4**.

Results for the seven eligible phase 3 trials are shown in **Table 2** [68–103]. Among these trials, patient age, gender, and ECOG PS were similar to those in the TARGET trial. Likewise, the predominant histology was clear cell type and most patients had undergone prior nephrectomy. However, MSKCC scores were considerably more heterogeneous. With the exception of AGILE 1051, all of the trials included a higher proportion of patients with intermediate or poor status than were included in TARGET (48%). MSKCC scores were also substantially worse in the expanded access AUS1 trial (**Table 1**) (not reported for EU-ARCCS and NA-ARCCS). Similar to TARGET, in which 98% of patients had ECOG PS ≤1, all patients in the phase 3 trials had ECOG PS ≤1 (where reported). In contrast, patients with ECOG PS 2 or higher were included in the EU-ARCCS and AUS-1 expanded access trials. Race was inconsistently reported, and no trial reported inclusion of more than 25% Asians.

In addition, phase 3 trials varied with respect to the number of prior systemic treatments administered. In TARGET, all patients’ tumors had progressed after one systemic treatment [1]. Sorafenib was also studied in the second-line in AXIS and INTORSECT. In AXIS 35% of patients had received prior cytokine treatment, 54% received prior sunitinib, 8% received prior bevacizumab, and 3% received prior temsirolimus treatment [74]. In INTORSECT, all patients received prior sunitinib treatment [76]. Two phase 3 trials, CROSS-J-RCC and SWITCH, examined sequential treatment approaches in which sorafenib was used following progression on first-line sunitinib and vice versa. For these trials, data were collected for both first- and second-line treatment, although data are not yet available for second-line treatment in CROSS-J-RCC [71,75]. In GOLD-RCC, patients received sorafenib third-line after disease progression on or within 6 months of the most recent of two prior therapies including one VEGF inhibitor and one mammalian target of rapamycin (mTOR) inhibitor [77,82,83]. In one of the phase 3 trials, all patients were treatment naive [68,69]. Finally, in the TIVO-1 trial, the patient populations were mixed with respect to the line of therapy [78–82].

**Fig 2:**
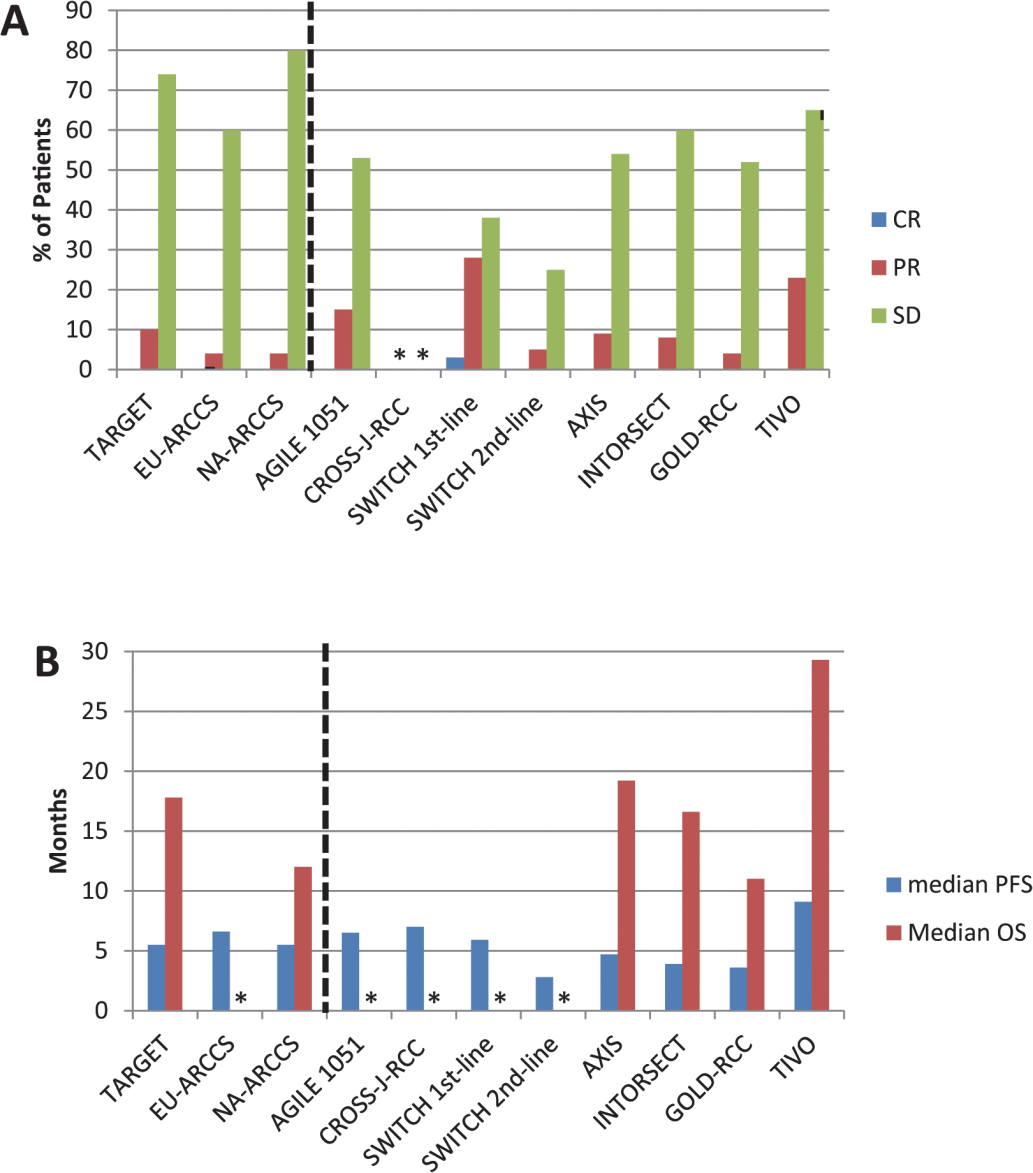
Sorafenib response rates, PFS, and OS in TARGET, associated expanded access trials, and subsequent phase 3 trials. A) Response rates. B) Median PFS and OS. Data for trials including patients for whom sorafenib was used ≥second line are indicated by stippling. *Data were not reported.

Median PFS ranged from 5.9 to 7.0 months in patients treated with first-line sorafenib (N = 341) [68–71]. In patients treated with second-line sorafenib (N = 691), PFS (95% CI) associated with sorafenib treatment ranged from 2.8 (not reported) to 4.7 (4.6–5.6) months [71–76]. In the third-line setting (N = 286), PFS (95% CI) was 3.6 (3.5–3.7) months [77]. Median PFS and OS for each of the phase 3 trials are represented graphically in **Fig. 2**, along with results from TARGET, EU-ARCCS, and NA-ARCCS.

**Fig 3:**
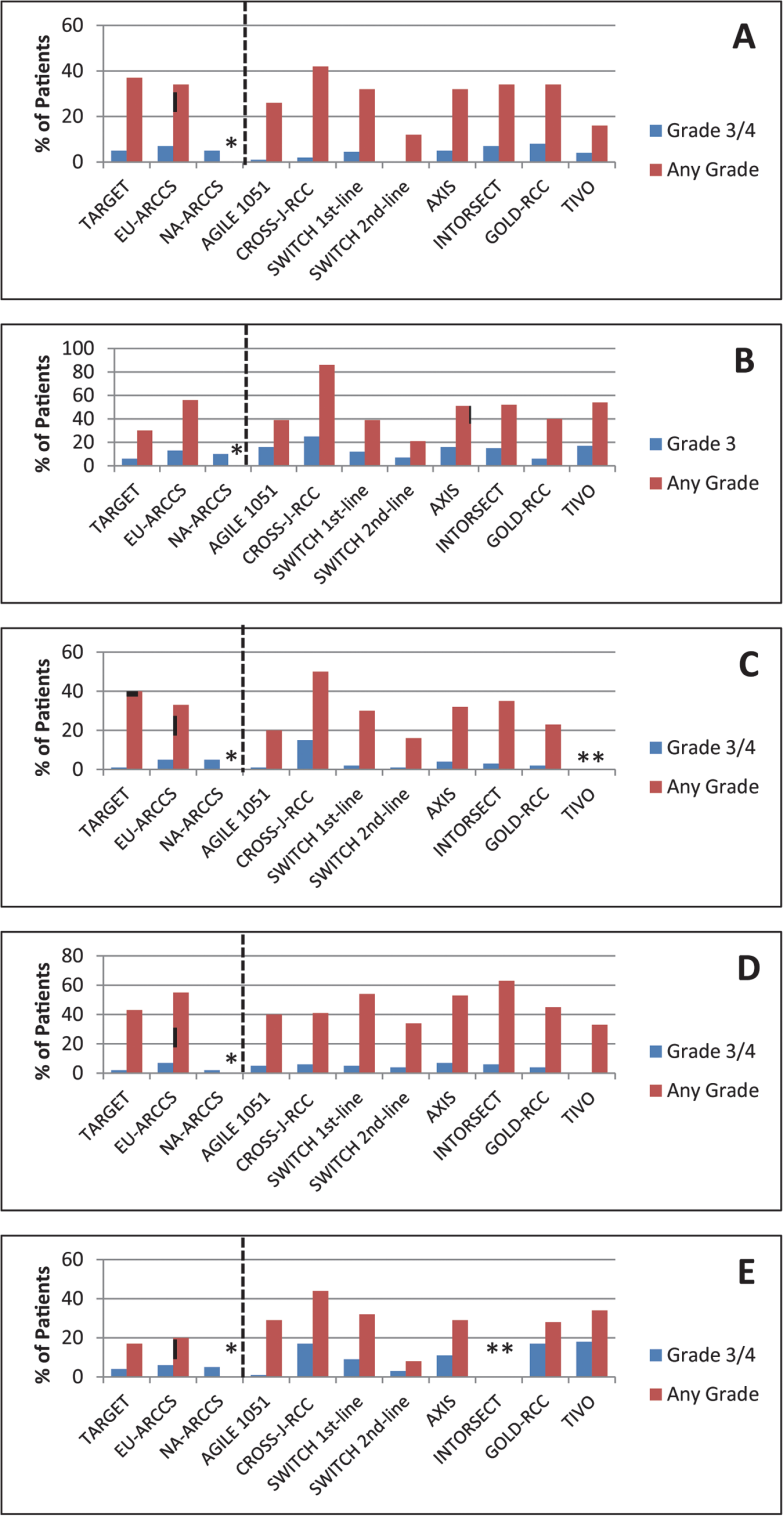
Incidences of select adverse events in TARGET, associated expanded access trials, and subsequent phase 3 trials. A) Fatigue; B) HFSR; C) Rash, desquamation; D) Diarrhea; E) Hypertension. Data for trials including patients for whom sorafenib was used ≥second-line are indicated by stippling. *Data were not reported.

Where reported, treatment-emergent AEs occurred in nearly all patients, and grade 3/4 AEs were seen in 36%-70%. Overall incidences of AEs by line of treatment are difficult to evaluate because data have not been reported for four of the seven trials. The incidences of serious AEs was reported for three trials and ranged from 25%-34%. Frequencies of select AEs occurring in >20% of patients in any phase 3 trial are detailed in **Table 2**. Fatigue, hand-foot skin reaction (HFSR), rash or desquamation, diarrhea, and hypertension were among the most frequent AEs. Incidences of these AEs are presented graphically in **Fig. 3** [68–103].

### Phase 2 trials

Published data were identified for a heterogeneous group of nine phase 2 trials that met eligibility criteria for this review (**Table 3**). First-line sorafenib was used in three randomized, open-label studies [25–30]. Median PFS (95% CI) ranged from 5.7 (5.0–7.4) to 9.0 (5.5–10.9) months. In a single-arm dose-escalation study in the first-line setting, patients received 400 mg twice daily (BID) for 4 weeks, and escalated to 600 mg BID for 4 weeks and finally to 800 mg BID, with response evaluated at 6 months. The dose-escalation protocol was tolerated by 18/67 patients [31,32]. The remaining patients had dose escalations and reductions as tolerated throughout the study. Overall median PFS (95% CI) was 7.4 (6.3–9.7) months. Subgroup analysis by dose (400, 600, or 800 mg BID) administered to patients for the longest duration showed median PFS (95% CI) of 3.7 (1.8–9.5), 7.4 (6.3–12) and 8.5 (5.6–14.9) months, respectively [32].

In three phase 2 trials, sorafenib was evaluated as second-line or later therapy in patients who had failed or progressed after cytokine therapy [35,36] or in patients refractory to sunitinib or bevacizumab [33] (**Table 3**).

One study [34] was a randomized, discontinuation trial in which patients with tumor growth or <25% tumor shrinkage during a 12-week run-in period of sorafenib treatment were randomized to receive continued sorafenib or placebo. Thirty-two patients received continued sorafenib; efficacy results are presented only for these patients. Safety results are presented for the entire enrolled population (N = 202), 91% of whom received prior interleukin (IL)-2, interferon, or nonspecified systemic anticancer therapy. Median PFS (95% CI) was, respectively, 4.4 (3.6–5.9) and 5.5 (not reported) months in patients (N = 79) previously treated with VEGF inhibitors [33,34], and 7.4 (6.5–13.1) and 7.9 (6.4–10.8) months in patients (N = 165) previously treated with cytokine therapy [35,36]. It should be noted that this particular study technically met inclusion criteria for this systematic review because it was published in 2006. However, efficacy data are reported only up to December 31, 2004.

Overall incidences of treatment-emergent AEs were not consistently reported, but ranged from 96%-100% where data are available [31,34,37]. Incidences of treatment-related AEs, where reported, ranged from 95%-97%, and grade 3/4 treatment-related AEs in the same studies ranged from 41%-69% [27,35]. Frequencies of the most common specific AEs are detailed in **Table 3**.

### Smaller studies and patient series

In addition to phase 2, phase 3, and expanded access trials, five small single-arm studies and three patient series reports were identified. Results for these studies are reported in **Table 4**. As mentioned above, one trial was listed as a phase 3 trial, but because it was not a randomized controlled trial and because only 39 patients were enrolled, it is included with this, more similar group of studies [60]. Notably, in two of the single-arm trials, median PFS [95% CI] was considerably longer than was seen in any of the phase 2/3 trials (9.5 [not reported] and 14 [0–31.7] months) [61,62]. These two studies were undertaken entirely in Chinese patients and 91% of the patients had received at least one prior systemic therapy.

## Discussion

Generally when a drug is demonstrated to have clinical utility and becomes approved for use, results from a pivotal phase 3 trial, and potentially one or a few earlier phase 2 studies, comprise the body of experience in terms of disease response and management of side effects. These studies therefore have a profound effect on drug labeling and its uptake and use in the community. Over time, clinical experience in management of dose- and course- limiting acute and chronic side effects typically matures, with positive impacts for both the patient experience and disease outcomes. However, information on the collective experience may not be readily available to the practitioner.

Sorafenib was approved for the treatment of RCC in 2005 after favorable PFS results were obtained in the pivotal TARGET trial. Since that time, a large number of studies using sorafenib in RCC therapy have been undertaken. The number of publications initially identified (2411) represents a dauntingly broad experience. The availability of published data from these studies provides an important opportunity to comprehensively evaluate how the safety and efficacy experience with this drug may have evolved. To that end, we have systematically reviewed the published literature for clinical studies conducted since 2005 that included the use of single-agent sorafenib for RCC.

In all, we identified 30 studies in which 2182 patients were treated with sorafenib. Among these, 1575 were treated in randomized controlled phase 3 trials. It is important to note that even among phase 3 trials, comparisons with TARGET should be undertaken with caution. Differences in trial design, patient baseline characteristics (including proportion of patients with higher-risk characteristics) duration of treatment and follow-up and reported endpoints have precluded quantitative comparisons or meta-analysis. There is no comprehensive patient-level database that spans those experiences, and no practical way to re-analyze response or toxicity assessments.

Whereas TARGET was a double-blind, placebo-controlled trial, all of the phase 3 trials (**Table 2**) identified in this review were open-label studies where the comparator arm was another targeted agent. Overall survival was the primary end-point in TARGET and PFS was the primary endpoint in the other seven phase 3 trials. Although it is not the intent of this review to provide a comparison with other agents, it is interesting to note that where reported, OS in sorafenib- treated patients was either similar to[58,61,86], or superior to[80][1] comparator agents.

Among the identified trials, patients differed with respect to baseline characteristics, perhaps most profoundly by MSKCC score and line of treatment. A recent retrospective review of control (comparator) arm data derived from clinical trials in RCC suggests that the characteristics of RCC patients at baseline have consistently improved over time and proposes that these differences may result from increased use of palliative nephrectomy, advances in surgical techniques, and earlier diagnosis [108]. The diversity of reported trial designs reflects not only evolving approaches in standard of care for RCC, but also the desire to further evaluate the role of sequential treatment for patients whose disease progresses during treatment with targeted agents that were unavailable at the time sorafenib was approved.

In the TARGET trial, patients’ tumors must have progressed after one systemic treatment within the previous 8 months. Sorafenib was used exclusively second-line for three other phase 3 trials. In contrast to TARGET, prior treatment in these trials consisted largely, if not entirely, of targeted non-cytokine therapies. As summarized in **Table 2** and **Fig. 2**, patients treated in the second-line setting appeared to have shorter PFS than in the 3 trials where patients were treated first-line with sorafenib, a finding that is in keeping with the trend towards poorer MSKCC score in the second-line trials. While the major impacts of risk-group, disease features, and line-of-treatment preclude a quantitative comparison, more contemporary data are generally similar to that seen in TARGET.

Disappointingly, safety data were inconsistently reported among the trials. Overall incidences of AEs were reported for five of the seven phase 3 trials included; Serious AEs were reported for only three studies. Incidences of grade 3/4 AEs varied substantially. In TARGET, overall incidences of AEs were reported only if they were considered treatment related. These and other factors, such as differing duration of treatment, preclude a meta-analysis with statistical characterization trends.

Specific treatment-emergent AEs reported in all phase 3 trials included in this review were largely similar to TARGET. However, whereas grade 3 HFSR was observed in 6% of patients in TARGET [1], it was reported in ≥15% patients in five of the more recent phase 3 trials [68,70,74,76,78,79,83]. Similarly, grade 3/4 hypertension occurred in 4% of patients in TARGET [1] and in 11%-18% in four of the more recent trials [70,74,75,77–79,103] (**Fig. 3**). One may speculate that some practice-related factors may be responsible for this. In more recent trials, increased familiarity with the use of sorafenib and its side effects may have reduced the frequencies of dose interruptions and reductions, resulting in overall higher dose intensity over the course of treatment. However, due to inconsistent reporting of dose intensities, this notion cannot be substantiated based on the reported data. As a bottom line for the clinician, the HSFR and hypertension issues seem more frequent in the current experience—an active management plan to mitigate remains an ongoing consideration.

Higher incidences of grade 3 HFSR and grade ≥3 hypertension were also reported in several of the phase 2 trials, although qualitative differences (treatment-emergent vs treatment-related AEs) and inconsistent reporting preclude determining the number of trials in which these occurred. Even with these rather small differences, it appears that the safety profile of sorafenib observed in the variety of patient populations and treatment settings studied is consistent with that observed in the TARGET trial, with no new signals.

For comprehensiveness, we included phase 2 and smaller trials in this review. Five of the phase 2 trials were randomized, and the remaining studies, including smaller trials and patient series listed in **Table 4**, were single arm. In addition to the limitations discussed above for the phase 3 trials, inherent potential bias in these trials should be considered when interpreting the results. Nonetheless, these trials may offer important insights. However, it is important to note that results reported for all of these studies may differ from those observed in daily clinical practice. Similarly, trends or absence of changes in reported prospective trials are not conclusively demonstrative of changes in daily clinical practice.

For example, among the seven phase 3 trials included in this review, none enrolled more than 25% of Asian patients. One phase 2 trial [35] enrolled exclusively Japanese patients and two smaller trials [61,62] and a patient series [58] report results in Chinese patients. PFS in these studies appeared longer than in TARGET or any other trial reported here (single-agent sorafenib results were not reported for the patient series). Yang et al. considered ethnic background as an important factor leading to these differences [61]. However, disease control rates and OS (where reported) did not show corresponding differences. These studies in Asian patients enrolled fewer than 100 patients each and did not include a control (non-Asian) population; additional studies would be necessary to clarify these findings. Incidences of AEs in Asian patients were similar to those observed in TARGET with the exception that grade 3 HFSR was higher in the smaller studies (**Table 4**), and treatment-related grade 3/4 hypertension was seen in 17% of patients in the phase 2 trial (**Table 3**). Understanding how ethnic features affect sorafenib efficacy or side effects remains a challenge.

In this review, we have comprehensively collected publications that describe the use of single-agent sorafenib in prospective studies in patients with RCC. The data are focused on the sorafenib experience, rather than comparative efficacy, with the goal of obtaining a broadened perspective on what to expect as medical practices evolve, beyond the database of the original pivotal trial. While the randomized controlled phase 3 trials likely provide the most robust information, important additional information may be gleaned from the inclusion of phase 2 and smaller studies, particularly as they may provide historical context or describe less well-represented populations. Comparisons among the included trials should be made with due caution as study design, patient populations, tumor characteristics, and prior drug exposure vary dramatically. Differences of patient characteristics a priori may be more important than differences of treatment plans. Notwithstanding the diversity of trial designs in this review, in examining the primary endpoints of this study, PFS and safety, we have observed no profound differences from the results observed in TARGET.

## Supporting Information

S1 Checklist PRISMA 2009 checklist complete. (DOC)
